# Flap-to-flap neovascularization for extended soft-tissue reconstruction of the upper-limb

**DOI:** 10.1016/j.jpra.2026.05.024

**Published:** 2026-05-15

**Authors:** Sébastien Durand, Célia Guttmann, Yves Harder

**Affiliations:** Department of Hand Surgery, Lausanne University Hospital and University of Lausanne, Avenue Pierre-Decker 5 1011 Lausanne, Switzerland

**Keywords:** Upper limb reconstruction, Soft-tissue defects, Necrotizing fasciitis, Anterolateral thigh flap, Groin flap

## Abstract

Extensive upper-limb soft-tissue defects may exceed the surface area achievable with conventional free flaps, making reconstruction challenging when durable coverage and functional preservation are required. We describe a novel reconstructive strategy to extend soft-tissue coverage based on secondary flap-to-flap neovascularization in which a pedicled flap achieves secondary vascular independence through neovascularization from the free flap skin paddle via the subdermal vascular plexus, thereby enabling delayed division without additional microsurgical anastomoses.

A 64-year-old man developed necrotizing fasciitis of the right upper limb following an insect bite. After repeated surgical debridements, a massive circumferential forearm defect extending to the elbow was associated with extensive tissue loss of the dorsal hand, first web space, and palm. A 45 × 11 cm chimeric anterolateral thigh–tensor fascia latae (ALT–TFL) free flap was harvested and anastomosed to the radial vessels, providing stable coverage of exposed extensor tendons and neurovascular structures. An ipsilateral pedicled 20 × 15 cm groin flap was inset onto the ALT skin paddle and subsequently divided, defatted, and wrapped around the thumb to recreate the first web space and resurface the palm.

At 18-month follow-up, soft-tissue coverage remained stable, with complete flap survival, no recurrence of infection, and good functional recovery.

Unlike sequential or conventional free-flap reconstruction, this approach does not require additional arterial inflow or flow-through anastomoses, relying instead on secondary flap-to-flap neovascularization. It may therefore represent a relevant strategy to extend reconstructive coverage beyond conventional flap dimensions in extensive upper-limb defects.

## Introduction

Necrotizing soft tissue infections, including necrotizing fasciitis, are uncommon but life-threatening conditions, with reported mortality rates ranging from 6 % to 76 %.^1^ These infections are characterized by rapid progression along subcutaneous tissues and fascial planes, frequently resulting in extensive necrosis. Early and repeated surgical debridement is mandatory but often results in large and complex soft tissue defects exposing tendons, muscles, neurovascular structures, and occasionally bone, eventually requiring advanced reconstructive strategies.

Although split-thickness skin grafts remain the most common method of defect reconstruction once infection control is achieved, it is often insufficient in extensive upper-limb defects when functional preservation and durable coverage are required. In selected cases, free tissue transfer becomes necessary to restore stable soft-tissue coverage and protect essential functional structures.^2^

The anterolateral thigh (ALT) flap is a reliable option for reconstruction of extensive upper-limb soft-tissue defects.^3^ Its coverage capacity may be further increased using a chimeric design incorporating a tensor fascia latae (TFL) fasciocutaneous component.^4^ Both components are supplied by branches of the lateral circumflex femoral artery (LCFA), allowing harvest of a large ALT–TFL chimeric flap based on a single vascular pedicle.^5^

However, even large chimeric free flaps may be insufficient in massive circumferential defects, and additional vascularized tissue may be needed to complete the reconstruction. We describe a novel flap-to-flap neovascularization strategy in which a secondary pedicled groin flap is inset onto the ALT’s skin paddle and subsequently survives through progressive neovascularization at the two flap’s interface (“parasitic flap”), thereby extending the flap’s dimension without requiring additional arterial inflow or flow-through anastomosis.

## Case report

We report the case of a 64-year-old man with a history of smoking and arterial hypertension who presented 48 h after an insect bite to the right hand with toxic shock syndrome. Clinical examination revealed hemorrhagic bullae, areas of skin necrosis and marked inflammatory edema of the hand. Repeated surgical debridements confirmed necrotizing fasciitis due to *Streptococcus pyogenes*. Intensive care management and antibiotic therapy were initiated, including Penicillin G (4 million IU four times daily) and intravenous clindamycin (900 mg three times daily).

**Figure 1:**
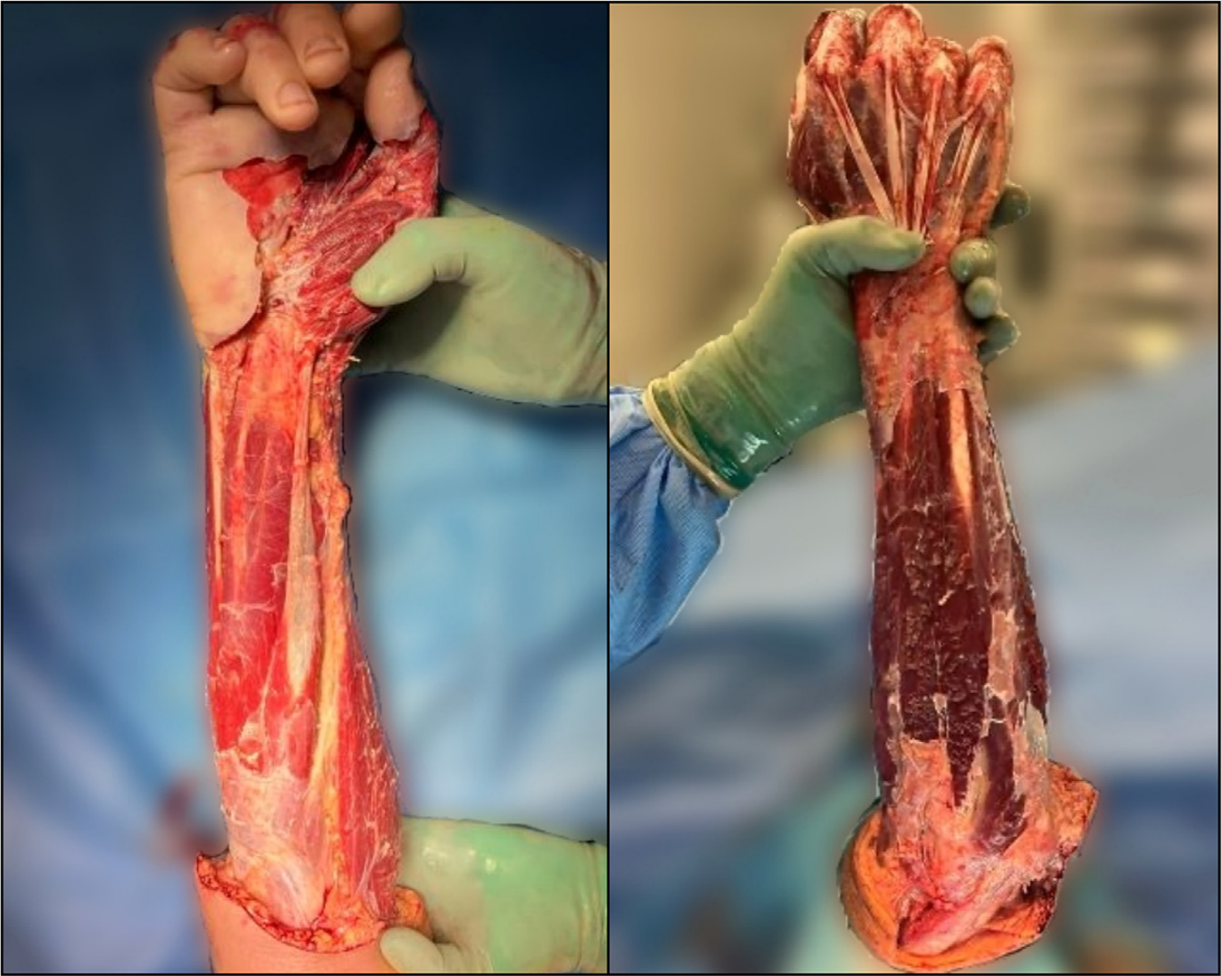
Volar and dorsal view of the large circumferential soft-tissue defect of the forearm extending from the elbow to the hand, including extensive involvement of the dorsal hand and first web space.

The patient sustained a massive circumferential soft-tissue defect of the entire forearm extending to the elbow, associated with extensive tissue loss of the dorsal hand, palm and first web space (Figure 1).

Initial coverage required a 45 × 11 cm flap to resurface the dorsal hand because of extensive exposure of the extensor tendons extending proximally to the elbow. Therefore, a chimeric fasciocutaneous ALT-TFL flap was harvested. Two dominant musculocutaneous perforating vessels were identified: one originating from the descending branch of the LCFA supplying the ALT component and one transverse branch from the LCFA supplying the TFL component. The vascular pedicle was dissected proximally until reaching the profunda femoris artery. The transverse branch of the LCFA ran beneath the femoral nerve; however, the skin paddle of the TFL could be pulled under the nerve during harvest, preserving pedicle continuity. An end-to-side anastomosis was performed between the LCFA pedicle and the radial artery. Veinous drainage was achieved using a superficial cephalic vein of adequate caliber, allowing reliable outflow and avoiding size mismatch. Particular attention was paid to flap positioning in order to avoid excessive tension or twisting of the pedicle. No signs of vascular compromise were observed during the postoperative course. The remaining forearm defect, particularly on the volar aspect was covered with a split-thickness skin graft.

**Figure 2:**
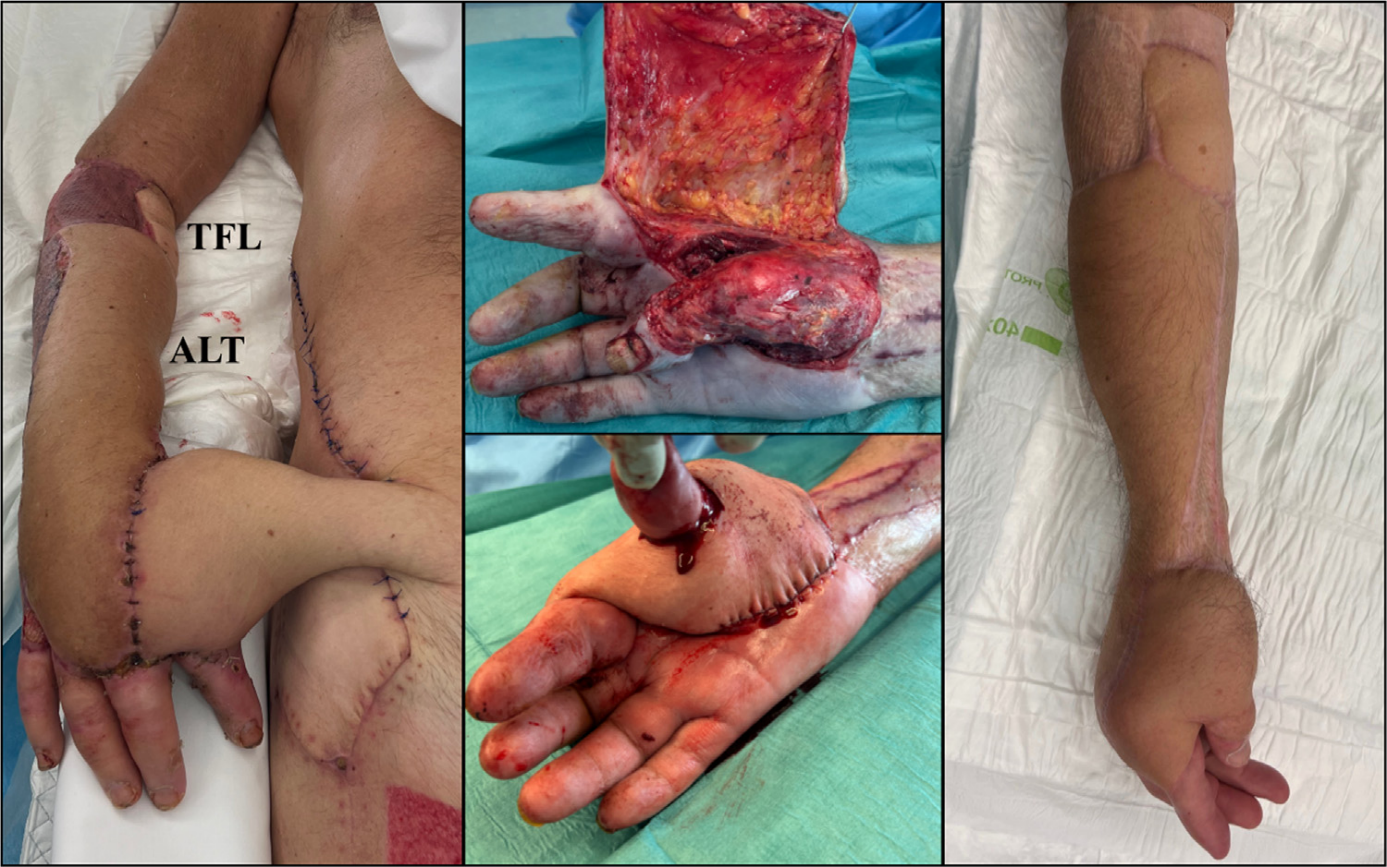
Intraoperative view showing the chimeric ALT-TFL free flap in place, with an ipsilateral pedicled groin flap inset onto the ALT skin paddle (left) to achieve flap-to-flap neovascular integration (“parasitic flap”). The scar visible inferior to the groin flap corresponds to the donor site of the combined ALT-TFL flap harvest. Second-stage appearance after division and defatting of the groin flap, followed by reconstruction of the first web space using a central slit incision to fashion a new commissure (middle). Clinical appearance at 18-month follow-up demonstrating stable soft-tissue coverage (right).

An ipsilateral pedicled groin flap was then raised and sutured onto the ALT skin paddle of the chimeric flap (Figure 2), contributing additional soft tissue coverage. After 3 weeks of progressive flap-to-flap neovascular integration, the groin flap was subsequently divided medially, defatted, and wrapped around the exposed thumb to recreate the first web space and to cover the palm defect, using a central slit to fashion a neocommissure (Figure 2). A three-week interval was chosen to allow sufficient neovascularization between the flaps, in accordance with established principles of flap integration.

**Figure 3:**
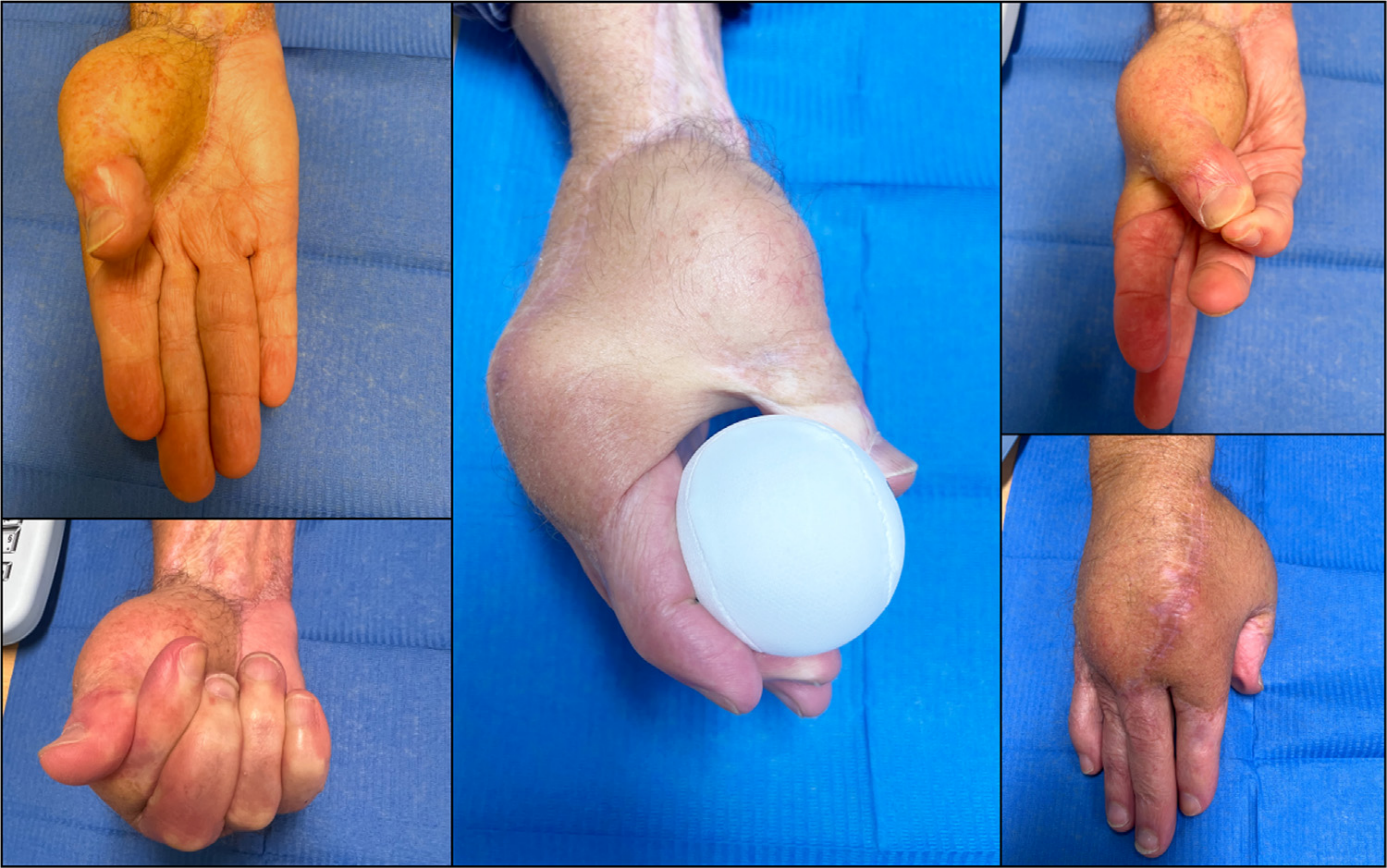
Final functional outcome at 18-month follow-up. Palmar view shows stable soft-tissue coverage. Functional grasp of a spherical object is preserved. Thumb abduction illustrates reconstruction of the first web space. Near-complete finger flexion is achieved and the dorsal view shows good flap integration and satisfactory skin quality.

At 18-month follow-up, soft-tissue coverage remained stable with complete flap integration and no evidence of recurrent infection (Figure 2). The patient reported no pain and regained independence in daily activities with mild residual finger stiffness (Figure 3).

## Discussion

Extensive upper-limb soft-tissue defects, particularly when associated with exposure of tendons, neurovascular structures, or bone, remain a major reconstructive challenge and frequently require complex soft tissue transfer other than skin grafts to restore both durable coverage and function.

Pedicled regional flaps such as the groin flap can provide reliable coverage for moderate size defects and have been described with dimensions reaching up to 35 cm in length and 15 cm in width. Larger composite regional solutions have also been reported, including combinations of bilateral groin flaps with abdominal flaps, allowing reconstruction of major defects of the forearm and hand.^6^

However, in cases of massive circumferential soft tissue loss of the forearm, such as in the present report, conventional pedicled options are often insufficient and may not allow adequate resurfacing of the entire defect. The survival of pedicled groin flaps depends on progressive neovascularization through the flap’s subcutaneous and subdermal plexus from adjacent healthy recipient skin. In circumferential defects of the forearm, where native skin is largely absent, this mechanism may be insufficient, rendering conventional pedicled options unreliable or technically unfeasible. Although a SCIP (superficial circumflex iliac artery perforator) flap could be considered in this anatomical region, it is primarily used as a free flap and is less suitable as a pedicled option for upper limb reconstruction. While staged pedicled flaps have been historically described, the present approach differs in that the pedicled flap is transferred onto a previously inset free flap, which serves as a vascularized recipient bed. This flap-to-flap neovascularization strategy represents a distinct reconstructive concept combining microsurgical and pedicled technique. At final follow-up, the patient achieved satisfactory functional recovery, with preserved hand mobility allowing performance of daily activities, supporting the clinical relevance of this approach.

In these situations, large free flaps such as the latissimus dorsi (LD), the anterolateral thigh (ALT), or extended rectus abdominis flaps (including boomerang-shaped designs) represent standard reconstructive choices.^7^ Nevertheless, even these “large” free flaps may occasionally fail to provide enough surface area to achieve complete coverage, potentially necessitating the use of a second free flap, either as a chimeric flap sharing the same vascular pedicle or as an independent free flap.

**Figure 4:**
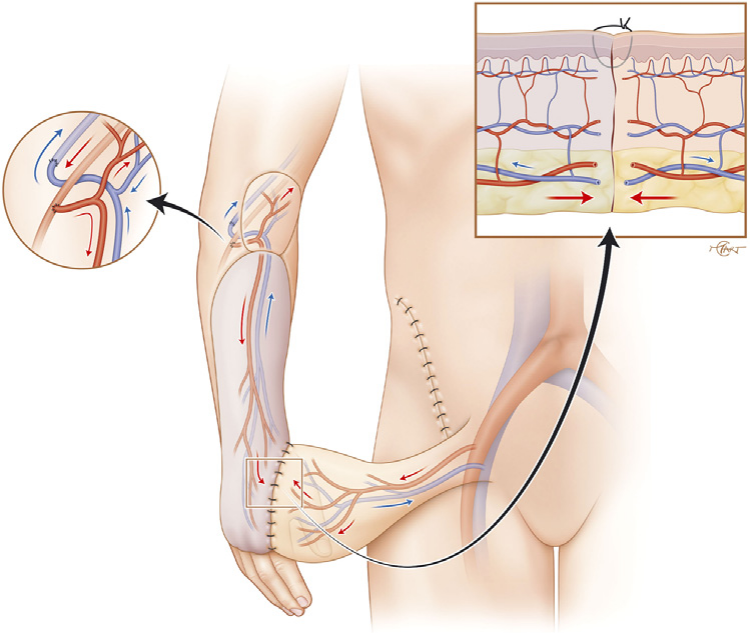
Initial flap to flap configuration. A pedicled groin flap is inset onto the skin paddle of a free ALT flap, allowing progressive neovascularization between the two flaps. Insets: left, microvascular anastomoses of the free flap to the radial vessels; right, dermal apposition at the suture interface without vascular connections.

**Figure 5:**
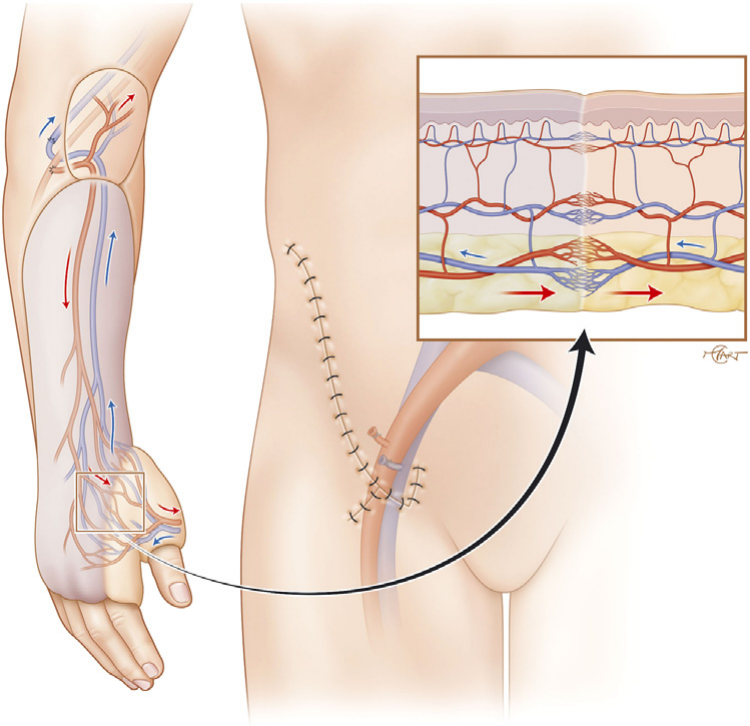
Flap division after neovascularization. The groin flap is divided from its native pedicle, surviving on ALT-derived perfusion. Inset showing mature subcutaneous and subdermal plexus vascular connections.

Accordingly, sequential or flow-through free-flap have been described as a solution for this type of defect,^8^^,^^9^ but such strategies require additional vascular in- and outflow anastomoses and may increase microsurgical complexity and operative risk. In contrast, the progressive flap-to-flap neovascularization relies on the combination of a well-integrated microvascular flap (ALT) that “hosts” the pedicled flap (groin flap) through progressive neovascularization of the two flap’s components without requiring additional microvascular anastomoses (Figure 4). The secondary pedicled flap, actually a groin flap requiring the ALT’s blood supply to survive after transection of its pedicle and therefore referred to as a “parasitic flap” (Figure 5). Classically, this mechanism occurs when the flap is applied to well-vascularized healthy native skin. In our case, however, the groin flap was successfully vascularized over the skin paddle of a free ALT flap, supporting the concept of flap-to-flap neovascular integration.

Although promising, this approach has important limitations. Flap-to-flap neovascularization requires sufficient time for progressive vascular integration, which may result in prolonged immobilization and delayed definitive reconstruction. This can be a significant drawback, particularly in upper-limb surgery where early mobilization is desirable.

Therefore, this technique should not be considered a standard alternative to conventional free flap reconstruction, but rather a complementary option in highly selected situations. It may be particularly useful in case of very large or circumferential defects exceeding the surface achievable with a single free flap, or when additional microsurgical anastomoses are not feasible or would increase surgical risk.

Careful patient selection is essential, and this approach should be reserved for situations in which conventional reconstructions options are insufficient or not feasible. In particular, it should be used with caution in patients with poor general condition, uncontrolled infection, or compromised local perfusion.

## Conclusion

This flap-to-flap vascularization strategy may represent a valuable option in highly selected cases of extensive upper-limb defects when conventional free flap dimensions are insufficient. However, its use should be carefully weighed against its limitations, particularly the need for staged reconstruction and prolonged immobilization.

## Ethical approval declaration

Our Institution (CHUV-Centre Hospitalier Universitaire Vaudois) does not require ethical approval for reporting case series (<or = 5).

## Informed consent declaration

Written informed consent was obtained from the patient for their anonymized information to be published in this article.

## Contributorship details

**Sébastien Durand:** Conceptualization, Methodology, writing – review & editing; **Célia Guttmann:** writing – review & editing; **Yves Harder:** review & editing.

## Declaration of generative AI and AI-assisted technologies in the writing process

This scientific manuscript has been primarily written by human authors, with the involvement of AI-generated technologies only for the purpose of shortening the text. While Generative Artificial Intelligence (AI) and AI-assisted technologies have been utilized to streamline the text and enhance its clarity, the substantive content, conceptualization, and interpretation have been exclusively carried out by human researchers.

## Funding

None.

## Declaration of competing interest

None declared

## References

[1] Ditsios K., Chitas K., Christidis P., Charatsis K., Katsimentzas T., Papadopoulos P. (2022). Necrotizing fasciitis of the upper extremity - a review. Orthop Rev (Pavia).

[2] Childers B.J., Potyondy L.D., Nachreiner R., Rogers F.R., Childers E.R., Oberg K.C., Hendricks D.L., Hardesty R.A. (2002). Necrotizing fasciitis: a fourteen-year retrospective study of 163 consecutive patients. Am Surg.

[3] Song Y.G., Chen G.Z., Song Y.L. (1984). The free thigh flap: a new free flap concept based on the septocutaneous artery. Br J Plast Surg.

[4] Hill H.L., Nahai F., Vasconez L.O. (1978). The tensor fascia lata myocutaneous free flap. Plast Reconstr Surg.

[5] Jaiswal D., Mantri M.R., Shankhdhar V.K., Wagh S.H. (2021). Chimeric ALT plus TFL perforator flap for breast reconstruction post radical mastectomy with large skin defect. Indian J Plast Surg.

[6] Gong Z., Tian D., Zhang J., Zhang G., Zhang B., Wang S., Liu J. (2009). Repair of large skin defect of forearm and hand using bilateral groin flaps and abdominal flaps]. Zhongguo Xiu Fu Chong Jian Wai Ke Za Zhi.

[7] Koul A.R., Nahar S., Prabhu J., Kale S.M., Kumar P.H. (2011). Free Boomerang-shaped extended Rectus Abdominis myocutaneous flap: the longest possible skin/myocutaneous free flap for soft tissue reconstruction of extremities. Indian J Plast Surg.

[8] Yoshimatsu H., Nakatsuka K., Karakawa R., Fuse Y., Yano T. (2024). The piggyback superficial circumflex Iliac perforator flap for complex free flap reconstructions. Plast Reconstr Surg Glob Open.

[9] Georgescu A.V., Corpodean A.A., Olariu O.D., Matei I.R. (2025). Flow-through procedure in Sequela after complex injuries of the hand with fingers. Amputation J Hand Surg Glob Online.

